# The need to protect older immigrants’ health in a changing policy landscape

**DOI:** 10.1093/haschl/qxag127

**Published:** 2026-05-23

**Authors:** Darrell J Gaskin, Kamaria Kaalund, Rachael R McCleary, Mervin Dino

**Affiliations:** Bloomberg School of Public Health, Johns Hopkins University, Baltimore, MD 21202, United States; Bloomberg School of Public Health, Johns Hopkins University, Baltimore, MD 21202, United States; Bloomberg School of Public Health, Johns Hopkins University, Baltimore, MD 21202, United States; Bloomberg School of Public Health, Johns Hopkins University, Baltimore, MD 21202, United States

**Keywords:** immigrant health, access to care, disparities, Medicare policy, Medicaid policy

## Abstract

**Introduction:**

There are approximately 8.6 million foreign-born older adults (aged 50 and older) in the United States. As immigrants age and transition from the labor force to retirement, maintaining their health and accessing to health care are major concerns. This study reports the extent to which older immigrants face barriers to care.

**Methods:**

This study uses 2019-2023 data from the National Health Interview Survey to determine whether foreign-born older adults have poor health status and face greater barriers to health care than native-born older adults.

**Results:**

We find that older foreign-born adults who are not US citizens are more likely to face challenges accessing health care. While they are less likely to report that they in fair or poor health, they are more likely to have not seen a doctor within the past year, not have a usual source care, and are more worried about paying for care if they become ill.

**Conclusion:**

These problems will likely worsen under current legislation, such as the 2025 federal budget reconciliation law, which enacted more exclusions to health insurance eligibility for certain immigration statuses. We offer recommendations that could help maintain access to care in the current political climate.

Key points

- Older immigrants have less access to care compared to older US-born adults.
- Older immigrants are concerned that if they become ill, they would not be able to afford medical treatment.
- Recent changes in federal health policy will likely worsen older immigrants’ access to care problems.

## Introduction

There are approximately 8.6 million foreign-born older adults (65+) in the United States according to 2023 data from the US Census Bureau's American Community Survey (ACS), accounting for 14.3% of the overall older adult population.^1^ This figure has grown steadily since the 1990s and is projected to continue to grow to nearly 25% in the next few decades.^2^ Older foreign-born adults in the United States represent a highly heterogeneous population, with variation in their countries of origin, reasons for migrating, English language proficiency, and financial status. Within this growing and diverse population, several barriers to health care utilization are evident. Exclusionary policies that restrict health insurance eligibility, the chilling effects of aggressive immigration enforcement, and persisting gaps in health insurance coverage, language accessibility, and health literacy challenge health care access among foreign-born older adults.

Medicare is the primary source of health insurance coverage for older adults aged 65 and above in the United States, providing access to hospitals, physicians, prescribed medications, and other medical services. However, eligibility for full Medicare benefits is limited to individuals who meet specific requirements and legal residency status. Prior to July 2025, lawfully present immigrants could qualify for Medicare by meeting the same age and work history requirements as US citizens (40 work quarters for Medicare Part A) or by being a lawful permanent resident (LPR) (ie, green card holder) who had continuously resided in the United States for 5 years. The 2025 H.R.1 legislation (P.L. 119-21) enacts restrictions on Medicare eligibility for foreign-born older adults, limiting access to health care for many noncitizens residing legally in the United States.^3,4^

This legislation prevents individuals who are lawfully present in the United States from becoming eligible for Medicare, Medicaid, and subsidized Marketplace coverage unless they are LPRs who meet the 5-year bar, Cuban/Haitian immigrants, or people residing under Compacts of Free Association (COFA) agreements. As a result, many immigrants who are lawfully present in the United States, but do not fall into the aforementioned categories, such as refugees and asylees without a green card, individuals with Temporary Protected Status, victims of trafficking, and survivors of domestic violence, among other noncitizen statuses, are excluded from eligibility for these programs or receiving federal subsidies. This legislation is projected to result in 1.4 million lawfully present immigrants losing health coverage by 2034, according to Congressional Budget Office (CBO) estimates. The CBO specifically estimates that the changes in Medicare eligibility restrictions for certain immigrants would lead to about 100 000 additional uninsured people per year, though it does not stratify these estimates by immigration status across all provisions, potentially underestimating the true impact.^5,6^

Beyond legally residing foreign-born individuals, under longstanding federal policy, undocumented immigrants are ineligible for federally sponsored health coverage. As such, access to health care coverage and care for undocumented immigrants in the United States continues to be severely limited, resulting in low health care utilization despite high rates of chronic disease and disabling conditions.^7,8^ Further, older foreign-born adults face significant health challenges, including mental health barriers, due to systemic discrimination, racism, and disparities in social determinants of health. These challenges are compounded by factors, such as poverty, xenophobia, language access, social cohesion, and exclusionary immigration policies driving these structural inequities.^9^ Furthermore, several empirical studies demonstrate a “chilling effect,” where spikes in anti-immigration policy enforcement discourages lawfully present immigrants, especially in mixed-status households, from using public benefits they are eligible for.^10,11^ Using data from the 2019-2023 National Health Interview Survey (NHIS), we examine whether older foreign-born adults have health problems, experience barriers to accessing health care, and how these challenges vary by citizenship status. In the midst of sweeping federal policy changes and restrictions to health coverage among lawfully present older adult immigrants, there is an important need to more rigorously examine differences in health outcomes by immigration status to guide mitigation strategies at the state and local levels, protect access to care, and support the safety net caring for this population.

### Data and Methods

Andersen's Behavioral Model of Health Services Use guided variable selections, identifying predisposing characteristics (eg, age, sex, race, education, marital status, citizenship status, and health beliefs), enabling resources (eg, insurance, income, and access to services), and need factors (both perceived and evaluated health status) as determinants of health care utilization.^12^ Using the National Center for Health Statistics (NCHS), NHIS, we explored the differences between general health status and health care access among older foreign-born adults relative to older US-born adults. For our study population, we pooled data from the 2019 to 2023 sample adult file, and the 2020 partial adult file, identifying 75 908 adults aged 50 and older.

### Outcomes

To measure general health status, and health care access and utilization among older adults, we focused on 5 outcomes. To measure general health status, we identified older adults who indicated having fair or poor general health. To measure health care access and utilization, we identified 4 outcomes indicating whether respondents (1) delayed medical care due to cost within the past 12 months, (2) had no doctor visit within the past year, (3) did not have a usual place to go for care, and (4) were “very worried” about paying medical bills if they get sick or have an accident. We relied on self-reported health status to avoid inadvertently conditioning outcomes on health care utilization, which is particularly important given our focus on health care access.

### Independent variables

Our independent variable of interest is the nativity and citizenship status of older adults. We identified those who were native born (ie, born in a US state or territory) and immigrants (ie, born outside of the United States). Among immigrants, we identified US citizens and non-US citizens (legal and undocumented residents). We then categorized older respondents based on their nativity and citizenship status into 3 groups: (1) foreign-born and *not* a US citizen, (2) foreign-born and a US citizen, and (3) native-born and a US citizen.

We controlled for age (50-64, 65-74, 75-84, 85+), sex, race (Asian non-Hispanic, Black non-Hispanic, Hispanic, White non-Hispanic, other/multiple race non-Hispanic), educational attainment (less than high school, high school, some college, bachelors, advanced), marital status (married, widowed, divorced, separated, never married, living with partner), income to federal poverty level ratio (<100%, 100%-124%, 125%-199%, 200%-399%, 400%+), health insurance status, US census region (Northeast, Midwest, South, West), and 2013 NCHS Urban–Rural Classification (Large Central Metro, Large Fringe Metro, Medium and Small Metro, Nonmetropolitan), and the survey year. Health insurance status is coded as a binary variable, and in NHIS, an individual is considered currently insured if they have coverage through private health insurance, Medicare, Medicaid, Children's Health Insurance Program (CHIP), military (TRICARE, Veterans Administration (VA) and CHAMP-VA), other state-sponsored health plans, or other government program. Models included dummy variables for survey year to adjust for temporal changes in population characteristics, health care access, and policy context across survey cycles.

### Statistical analysis

We used descriptive frequency and percentages to observe the association between the nativity and citizenship status of older adults and their health status, health care access and utilization outcomes, and independent predictor characteristics. The independent variable of interest is a 3-categorical variable: foreign-born noncitizen, foreign-born citizen, and US-born (reference category). Differences between the groups were assessed using Pearson's chi-square test. Due to the prevalence of the outcome variables, modified Poisson regression models were used to test the association between the nativity and citizenship status of older adults and health status, health care access, and utilization. We estimated unadjusted and adjusted models controlling for our independent variables. We stratified the analysis by age, estimating models for 50-64-year-old adults and 65 and older adults separately, to compare the foreign-born adults to US-born Medicare and non-Medicare eligible adults separately. Survey estimation procedures were employed for the analysis due to the complex survey design of NHIS. Data construction and analysis were done using Stata/SE 18 (StataCorp, College Station, TX, USA).

### Limitations

The limitations of this analysis should be considered when interpreting the results. First, the NHIS relies on self-reported information, which may be subject to recall bias. Second, we acknowledge potential limitations related to the measurement of citizenship status in the NHIS. Citizenship is self-reported and may be subject to misclassification if respondents are reluctant to disclose noncitizen status. Such underreporting is most likely to occur among noncitizens, which would bias our estimates toward the null and likely lead to conservative estimates of disparities between noncitizen and citizen older adults. Due to these reliability concerns, it is important that these estimates, and any estimates related to immigration status and health care, be interpreted with caution. Third, there is no data available on the immigration status of individuals within the noncitizen category; thus, we were unable to identify how these trends vary across more specific subgroups. Fourth, due to the Coronavirus Disease 2019 (COVID-19) pandemic, NHIS switched from conducting in-person interviews to phone interviews, which affected response rates from 2020. Fifth, this is a cross-sectional data analysis; therefore, we are not estimating causal relationships between citizenship status and health outcomes.

Sixth, we did not include a measure of duration of US residence in the present analysis; therefore, residual heterogeneity among immigrant subgroups may remain. Finally, we are unable to control for unmeasured potential confounders (eg, language access and proficiency, extent of acculturation, and provider characteristics).

## Results

The pooled analytic sample included 76 193 adults aged 50 and older. Approximately 5% (*n* = 2573) were foreign-born noncitizens, 12% (*n* = 7887) were foreign-born citizens, and 83% (*n* = 65 733) were native-born citizens (Table 1). Across health-related outcomes, foreign-born older adults reported higher rates of fair or poor health, delays in medical care, no doctor visit in the past year, no usual place for care, and worry about paying medical bills if injured or sick.

**Table 1.** Descriptive statistics by foreign-born and citizenship status for older adults (50+).

	Total (*N, %)*	Foreign-born noncitizen	US-born	Foreign-born citizen	*P*-value
	76 193 (100.0)	2573 (5.0)	65 733 (82.6)	7887 (12.4)	
Outcome, %					
Fair/poor health	20.8	28.4	20.5	20.1	<0.001
Delayed medical care due to cost	5.7	11.6	5.6	4.4	<0.001
No doctor visit within past year	9.1	18.3	8.5	9.5	<0.001
No usual source of care	4.6	12.7	4.1	4.0	<0.001
Very worried about paying medical bills if sick or in accident	13.3	36.5	10.9	19.6	<0.001
Covariates, %					
Age category					<0.001
50-64	52.8	72.8	51.2	55.5	
65-74	28.2	18.3	29.1	26.2	
75-84	14.2	6.6	14.8	13.2	
85+	4.8	2.3	5.0	5.1	
Female	53.0	52.3	53.0	53.6	0.564
Race					<0.001
White—non-Hispanic	71.0	13.4	81.4	24.9	
Black—non-Hispanic	10.4	5.5	10.8	9.8	
Hispanic	11.7	63.5	5.2	34.0	
Asian—non-Hispanic	5.1	16.7	0.6	30.2	
Other/multiracial—non-Hispanic	1.8	0.8	2.0	1.4	
Education					<0.001
Less than high school	12.5	48.1	9.0	21.5	
High school	28.0	23.8	29.1	22.8	
Some college	28.6	11.3	30.8	21.1	
Bachelors	18.3	10.5	18.5	19.6	
Advanced	12.7	6.3	12.7	15.1	
Marital status					<0.001
Married	61.5	66.1	60.0	70.0	
Widowed	12.2	9.7	12.6	10.3	
Divorced	13.4	6.7	14.3	10.4	
Separated	1.3	4.8	1.0	2.0	
Never married	7.4	6.9	7.8	5.0	
Living with partner	4.2	5.8	4.4	2.5	
Poverty category					<0.001
Negative (<100%)	8.5	19.6	7.5	10.3	
Near poor (100%-124%)	3.8	8.1	3.4	4.8	
Low income (125%-199%)	13.7	22.5	12.8	15.9	
Middle income (200%-399%)	29.6	31.1	29.5	29.4	
High income (400%+)	44.5	18.6	46.7	39.6	
Have health insurance	95.7	76.5	96.8	96.4	<0.001
Region					<0.001
Northeast	18.4	14.9	17.8	23.6	
Midwest	21.3	7.9	23.8	9.6	
South	38.0	37.4	38.9	32.1	
West	22.4	39.8	19.5	34.7	
2013 NCHS Urban-Rural Classification					<0.001
Large Central Metro	27.5	52.0	22.5	50.5	
Large Fringe Metro	25.1	18.7	25.2	26.8	
Medium and Small Metro	31.2	23.4	33.5	19.2	
Nonmetropolitan	16.3	6.0	18.8	3.5	

Source: Authors’ analysis of data from the National Health Interview Survey, 2019-2023.

Decimals rounded to the nearest tenths place. Values are presented as weighted population estimates (*N*) and weighted percentages (%). Estimates account for the complex survey design and are nationally representative.

Compared with US-born older adults and foreign-born naturalized older adults, foreign-born noncitizen older adults were disproportionately younger, were more likely to be Hispanic, had lower incomes, and had lower levels of educational attainment (see Table 1). Compared to their counterparts, foreign-born older adults had the lowest rates of health insurance coverage (76.5% vs approximately 96% for both US-born and naturalized older adults). Foreign-born older adults were concentrated in large metro areas. Among US-born older adults, 22.5% resided in large central metro areas compared to 52.0% among noncitizens and 50.5% among naturalized citizens.

Results from the adjusted modified Poisson regression model (Table 2) show that foreign-born noncitizen older adults had a 12% lower prevalence (prevalence ratio [PR] = 0.88; 95% CI, 0.80-0.96) of reporting fair or poor health, and foreign-born naturalized older adults had a 15% lower prevalence (adjusted PR [aPR] = 0.85; 95% CI, 0.79-0.91) of reporting fair or poor health compared to their US-born counterparts. Foreign-born noncitizen older adults were significantly more likely to report not seeing a doctor within the last year (aPR = 1.21; 95% CI, 1.07-1.38), not having a usual place to go for care (aPR = 1.44; 95% CI, 1.21-1.72), and that they were very worried (aPR = 1.33; 95% CI, 1.19-1.48) about paying medical bills. Foreign-born naturalized citizens were more likely to report being very worried (aPR = 1.42; 95% CI, 1.30-1.54) about paying medical bills if they became sick or were injured in an accident. However, they were no more likely to report not seeing a doctor within the last year or not having a usual place to go for care than US-born older adults. The associations of the control demographic and socioeconomic variables are in the expected directions (see Appendices Tables S1-S3).

**Table 2.** Association between fair or poor health and health care access problems and citizenship status of older foreign-born adults by age group.

	Unadjusted model		Adjusted model	
Outcome	Foreign-born noncitizen^a^	Foreign-born citizen^a^	Foreign-born noncitizen	Foreign-born citizen
	Prevalence rate [CI]	Prevalence rate [CI]	Prevalence rate [CI]	Prevalence rate [CI]
Aged 50+				
Fair/poor health	1.38 [1.27, 1.50]***	0.98 [0.92, 1.05]	0.88 [0.80, 0.96]**	0.85 [0.79, 0.91]***
Delayed medical care due to cost	2.08 [1.80, 2.42]***	0.79 [0.68, 0.92]**	0.91 [0.76, 1.10]	0.85 [0.72, 1.01]
No doctor visit within past year	2.18 [1.96, 2.43]***	1.11 [1.01, 1.22]*	1.21 [1.07, 1.38]**	1.06 [0.96, 1.18]
No usual source of care	3.05 [2.69, 3.46]***	0.96 [0.83, 1.10]	1.44 [1.21, 1.72]***	1.00 [0.85, 1.18]
Very worried about paying medical bills if sick or in accident++	3.34 [3.10, 3.60]***	1.80 [1.68, 1.93]***	1.33 [1.19, 1.48]***	1.42 [1.30, 1.54]***
Aged 50-64				
Fair/poor health	1.05 [0.95, 1.15]	0.83 [0.95, 1.15]***	0.84 [0.74, 0.95]**	0.79 [0.70, 0.88]***
Delayed medical care due to cost	1.44 [1.31, 1.58]***	0.72 [0.64, 0.80]***	0.88 [0.71, 1.10]	0.85 [0.70, 1.03]
No doctor visit within past year	1.59 [1.51, 1.68]***	0.94 [0.89, 1.00]*	1.20 [1.04, 1.38]*	1.05 [0.93, 1.19]
No usual source of care	2.00 [1.88, 2.13]***	0.75 [0.69, 0.82]***	1.33 [1.09, 1.62]**	0.96 [0.79, 1.18]
Very worried about paying medical bills if sick or in accident++	2.68 [2.55, 2.81]***	1.50 [1.41, 1.59]***	1.37 [1.22, 1.53]***	1.41 [1.28, 1.56]***
Aged 65-85+				
Fair/poor health	1.55 [1.37, 1.78]***	1.17 [1.09, 1.27]	0.94 [0.83, 1.06]	0.92 [0.85, 1.00]*
Delayed medical care due to cost	3.23.06 [2.42, 4.32]***	1.00 [0.77, 1.30]	1.35 [0.91, 1.99]	0.83 [0.59, 1.18]
No doctor visit within past year	2.06 [1.59, 2.67]***	1.13 [0.95, 1.33]*	1.28 [0.93, 1.76]	1.05 [0.86, 1.28]
No usual source of care	3.14 [2.32, 4.26]***	1.03 [0.81, 1.31]	1.87 [1.21, 2.89]**	1.08 [0.79, 1.47]
Very worried about paying medical bills if sick or in accident^b^	4.08 [3.51, 4.73]***	2.24 [2.01, 2.50]***	1.51 [1.24, 1.84]***	1.37 [1.18, 1.60]***

Source: Authors’ analysis of data from the National Health Interview Survey, 2019-2023.

Adjusted modified Poisson models controlling for age, sex, race, education level, marital status, poverty status, insurance possession, region, urban/rural metro status, and survey year. Decimals are rounded to the nearest hundredths place. Estimates account for the complex survey design and are nationally representative.

^a^Reference category: US-born.

^b^Reference category: somewhat worried or not at all worried.

^*^ *P* < 0.05; **P < 0.01; ***P < 0.001.

Age-stratified analyses suggested that the association between citizenship status and fair/poor health differed by age group. Similar to the unstratified findings, foreign-born noncitizens and foreign-born citizens aged 50-64 demonstrated lower adjusted prevalence of fair/poor health relative to US-born older adults (aPR = 0.79; 95% CI, 0.70-0.88 and aPR = 0.84; 95% CI, 0.74-0.95, respectively). Among foreign-born noncitizens and foreign-born citizens aged 65 and older, this association was attenuated with estimates moving closer toward the null. The association was only marginally statistically significant for foreign-born citizens aged ≥65 years.

In age-stratified analyses, foreign-born noncitizens maintained higher prevalence of delayed medical care, going a year without seeing a doctor, not having a usual source of care, and being worried about medical bills than foreign-born citizens, relative to US-born older adults.

## Discussion

Our findings illuminate disparities in health status and health care access among older adults in the United States. At the population level, older foreign-born adults experience substantially worse health than US-born older adults, reflecting the lived structural disadvantage associated with immigration status. However, after adjusting for sociodemographic, socioeconomic, and geographic factors, foreign-born older adults demonstrated lower adjusted prevalence of fair/poor health compared to US-born older adults, despite reporting greater worry related to the cost of care, reduced access, and reduced utilization. These disparities were more pronounced for foreign-born older adults without citizenship status. Among older adults aged 50-64, foreign-born citizens show a clearer health status advantage relative to US-born adults, while foreign-born noncitizens appear to show a weaker advantage or less favorable health. Among those aged 65 and older, these differences attenuate, and the slight advantage demonstrated by foreign-born citizens is only marginally significant.

Foreign-born noncitizen older adults exhibit significantly greater barriers to health care relative to US-born older adults, they are less likely to report having a usual source of care and regularly seeing a doctor, and more likely to report that they were very worried about their ability to pay medical bills. Citizenship status appears to serve a protective role for health status and access among older adults. We hypothesize this is because citizenship directly mitigates some structural barriers to health care by providing pathways to safety net eligibility and insurance coverage.

Prior work elucidates this dual phenomenon in which immigrants face significant barriers to accessing and affording care yet often exhibit and report better overall health outcomes than their US-born counterparts for a period of time before converging in later life.^13,14^ While some data highlight significant health burdens and chronic disease prevalence among foreign-born adults compared to US-born adults, particularly for those in lower-income groups,^13,15^ other research points to an immigrant health advantage (also known as the immigrant health paradox).^16-19^

Potential explanations for this advantage include selection bias where healthier individuals immigrate to the United States and sicker individuals emigrate back to their home countries, protective factors from one's origin culture (eg, social support, family cohesion, and collectivism), or errors in data reporting and quality.^20^ Also, international comparisons show that Americans have worse health outcomes than residents of other high-income nations, with shorter life expectancies and higher rates of chronic disease.^21,22^ This phenomenon, described as the “US health disadvantage,” points to structural policies and features of life in the United States that contribute to poor health outcomes.^23^

The COVID-19 pandemic may have amplified health care access disparities observed in this study. Individual and system-level barriers to health care access worsened during the COVID-19 pandemic for immigrant populations in the United States.^24^ Observed differences in health status and health care access may reflect health care disparities that were intensified by the pandemic, particularly for minoritized immigrant populations. What is clear is that the directionality of health outcomes (ie, favorable or adverse) and health care utilization patterns of foreign-born adults depend on social, cultural, economic, and political factors as well as age of immigration and country of origin.^25,26^

Our findings are consistent with other studies exploring health trends among adult immigrants in the United States and demonstrate the role immigration status plays on shaping access to health care.^27^ Beyond immigration status, immigration policies, more broadly, are key drivers of health inequities.^28^ In January 2025, federal policy protections that previously restricted federal immigration authorities’ presence at “sensitive locations,” including hospitals, schools, and churches, were rescinded, removing uniform nationwide limits on enforcement in these settings. A KFF report from earlier this year found that 14% of all immigrants and nearly 50% of likely undocumented immigrant adults within this group reported avoiding seeking medical care since January 2025 due to immigration-related concerns.^29^ The expansion of federal immigration operations since this period has increased raids in sensitive areas, exacerbating widespread immigrant fears related to seeking health care, even among those with legal status, including citizens, legal permanent residents.^30^

For dual eligible foreign-born adults, barriers to care are increasingly worrisome. Foreign-born adults dually eligible for Medicare and Medicaid already experience significant health disparities, including higher rates of chronic disease, functional limitations, and higher rates of disability compared to nondually eligible foreign-born adults.^31^ When these disparities in health challenges are coupled with barriers resulting from exclusionary policies, the lack of culturally and linguistically inclusive care only worsens health care access for foreign-born adults. Low-income foreign-born adults between the ages of 50 and 64 will also face greater barriers to care. Beginning in January 2027, many individuals enrolled in Medicaid, aged 19-64, will need to satisfy new requirements to maintain their coverage, such as completing 80 hours of work, school, or community engagement a month and requiring more frequent eligibility redeterminations, which significantly increases the risk of coverage loss due to administrative hurdles. Coverage losses among older foreign-born adults in the coming years will disrupt prevention services and chronic disease management, restrict home and community-based services that support daily functioning, and worsen financial pressures, contributing to medical debt and increasing economic instability among immigrant families, including mixed-status households.

Prior to this legislation, several states have attempted to mitigate federal restrictions through state-funded coverage programs and targeted expansions (eg, California, Illinois, and New York).^32^ However, new federal penalties and fiscal pressures are limiting their ability to sustain or scale these efforts, and in some cases, states have sunsetted these state-funded coverage programs, leaving many foreign-born older adults with fewer options for health coverage. At the same time, other states, including Texas and Florida, have adopted measures requiring hospitals to collect information on patients’ immigration status, potentially exacerbating fear and reducing care utilization.^33^

Recent immigration policy and enforcement activities further deteriorate already fragile pathways to health care for older adult immigrants in the United States. The Trump Administration has targeted sanctuary jurisdictions for militarized immigration enforcement, with heightened risk for hospitals and health care facilities and provoking widespread fear within local communities.^13,34^ These factors disproportionately impact older adult immigrants who are enrolled in Medicaid, individuals awaiting status changes, undocumented immigrants, those with Temporary Protected Status, and other newcomers. These barriers underscore the need for more granular data on older adult immigrants and future research examining differential health care access among policy-relevant subgroups.

Given the magnitude of these policy impacts on older adult immigrants and noncitizens, we highlight 3 near-term, systems-level recommendations/calls to action for state and local governments, practitioners, health care providers, and academic institutions that focus on harm mitigation, protecting access to care, and supporting the broader US safety net. We rightfully acknowledge that these recommendations will not be possible for certain jurisdictions, and a deeper feasibility study of these recommendations falls outside the scope of this article. However, we find it important to briefly discuss state and local strategies and community-level approaches to protect access to care and health-promoting resources for older adult immigrants, with mitigating harm as central to the approach.

First, immigrant-serving organizations, including hospitals, health care providers, and community-based organizations, should work in coalition with state and local policymakers and advocacy groups to ensure that guidance on health insurance eligibility restrictions under H.R.1, particularly for Medicare and Medicaid, reach immigrant families and their providers. Hospitals and health care providers can adopt procedures and staff education to promote consistent, reliable, and tailored messaging to affected patients and caregivers. State and local governments, including health departments, Medicaid, and human service agencies, can work together on coordinated communications outreach to support safety net providers, including federally qualified health centers (FQHCs), free and charitable clinics, and community organizations serving older adult immigrants and noncitizens impacted by these policy changes. At the neighborhood level, safety net providers can support and build rapid response networks and other urgent response models that promote cross-collaboration during immigration emergencies and facilitate navigation to legal resources.^35^

Second, safety net providers should designate immigrant health liaisons responsible for partnership building, communications, and operations to promote organizational readiness. Immigrant health liaisons should work with local community partners and subject matter experts to implement model practices that ensure health care facilities are prepared for an influx of uninsured, presence of federal immigration authorities, and how to safeguard protected health information against unlawful information sharing with federal agencies for the purposes of immigration enforcement.^34^ Designated immigrant health liaisons may also be responsible for facilitating readiness training for frontline staff on how to respond to visits from federal immigration authorities, building processes to navigate immigrant patients, including those experiencing enrollment losses, to health-promoting resources, garnering support from organizational leadership, and ensuring operational procedures promote welcoming and inclusive spaces for immigrant patients. These types of roles are essential to promoting accurate and credible information sharing and building trust between immigrant communities and institutions.

Third, safety net providers and organizations serving older adult immigrants and noncitizens should disseminate accurate and multilingual Know Your Rights resources, including resources specific to immigrant eligibility in health coverage and food assistance programs.^36^ Given the projected Medicare enrollment losses among lawfully present older adult immigrants and noncitizens, hospitals, especially, should provide options for financial assistance and equitable navigation supports for patients experiencing disruptions in coverage and care. States such as Colorado, Illinois, Maryland, and New Mexico have recently implemented laws requiring hospitals to put procedures in place (eg, screening all patients for financial assistance eligibility) that protect uninsured individuals from medical debt.^37^ The need for similar state-level policy interventions that protect all uninsured individuals, including older immigrant and noncitizen adults excluded from health insurance coverage, will continue to increase concomitant with projected coverage losses under H.R.1.

Given barriers to citizenship, health coverage, and health care among older adult immigrants and noncitizens, it is critical that sensible and equitable responses are pursued. Without effective structural and multilevel interventions, the barriers identified in the current study are bound to worsen as H.R.1 provisions are implemented and lawfully present older adult immigrants begin to experience enrollment losses and greater restrictions to care and supports. These drastic enrollment losses may also create rippling effects on the US safety net system, with reduced access to preventative services, greater reliance on hospital emergency departments, and increases in uncompensated care among the US older adult immigrant population.

Further studies are needed to evaluate these effects and mitigation strategies. Contextualizing our study's findings in the current political climate and H.R.1 implementation, we can infer that older adult foreign-born noncitizens will continue to experience barriers related to access to health care, affordability, timeliness of care, language access, cultural responsiveness, and other factors—if not addressed at multiple intervention points. Future research should explore the intersections of exclusionary immigration policies and health outcomes and health care utilization among this population. In addition, practitioners, policymakers, and academia should work together on new approaches to protecting the health of immigrant and noncitizen communities and mitigating the cascading negative effects of recent federal policy changes.

## Supplementary Material

qxag127_Supplementary_Data
