# Supplementary material for: The need to protect older immigrants’ health in a changing policy landscape
Source: Health Aff Sch. 2026 May 23;4(6):qxag127. doi: 10.1093/haschl/qxag127 (PMC13268766; doi:10.1093/haschl/qxag127)
Supplement: qxag127_Supplementary_Data [file qxag127_supplementary_data.zip › Appendix Tables A1-3.docx]

Appendix Table A1. Association between Fair or Poor Health and Health Care Access Problems and the Sociodemographic Characteristics of the Older Adults

| Outcome | Fair/Poor Health | Delayed medical care due to cost | No doctor visit within past year | No usual place to go for care | Very worried about paying medical bills if sick or in accident† |
| --- | --- | --- | --- | --- | --- |
|  | Prevalence Rate [CI] | Prevalence Rate [CI] | Prevalence Rate [CI] | Prevalence Rate [CI] | Prevalence Rate [CI] |
| Sociodemographic Characteristics |  |  |  |  |  |
| Age Category |  |  |  |  |  |
| 50-64 | [Ref] | [Ref] | [Ref] | [Ref] | [Ref] |
| 65-74 | 1.05 [1.00,1.09]* | 0.44 [0.40,0.48]*** | 0.56 [0.52,0.60]*** | 0.64 [0.58,0.71]*** | 0.54 [0.51,0.57]*** |
| 75-84 | 1.20 [1.14,1.26]*** | 0.22 [0.18,0.26]*** | 0.30 [0.27,0.34]*** | 0.38 [0.33,0.45]*** | 0.38 [0.35,0.42]*** |
| 85+ | 1.38 [1.30,1.47]*** | 0.15 [0.10,0.21]*** | 0.33 [0.27,0.40]*** | 0.37 [0.29,0.47]*** | 0.33 [0.28,0.39]*** |
| Female | 0.91 [0.88,0.94]*** | 1.29 [1.20,1.38]*** | 0.69 [0.66,0.73]*** | 0.58 [0.53,0.63]*** | 1.19 [1.13,1.25]*** |
| Race |  |  |  |  |  |
| White Non Hispanic | [Ref] | [Ref] | [Ref] | [Ref] | [Ref] |
| Black Non Hispanic | 1.13 [1.07,1.19]*** | 0.84 [0.75,0.95]** | 0.66 [0.59,0.74]*** | 0.62 [0.53,0.73]*** | 1.21 [1.12,1.31]*** |
| Hispanic | 1.15 [1.08,1.22]*** | 0.93 [0.80,1.09] | 0.93 [0.84,1.03] | 0.85 [0.71,1.00] | 1.44 [1.32,1.58]*** |
| Asian Non Hispanic | 0.98 [0.87,1.10] | 0.58 [0.44,0.78]*** | 1.17 [1.00,1.36]* | 0.98 [0.76,1.27] | 1.02 [0.89,1.17] |
| Other/Multi-race Non Hispanic | 1.22 [1.08,1.39]** | 1.00 [0.77,1.30] | 0.94 [0.78,1.13] | 1.12 [0.85,1.46] | 1.09 [0.91,1.30] |
| Education |  |  |  |  |  |
| Less than High School | 1.29 [1.23,1.35]*** | 1.13 [1.00,1.28]* | 0.93 [0.84,1.02] | 1.09 [0.95,1.26] | 1.09 [1.02,1.18]* |
| High School | [Ref] | [Ref] | [Ref] | [Ref] | [Ref] |
| Some College | 0.88 [0.84,0.92]*** | 1.09 [1.00,1.19] | 0.90 [0.84,0.97]** | 0.95 [0.85,1.05] | 0.94 [0.88,0.99]* |
| Bachelors | 0.60 [0.56,0.64]*** | 0.96 [0.85,1.09] | 0.95 [0.87,1.03] | 0.98 [0.87,1.10] | 0.78 [0.71,0.84]*** |
| Advanced | 0.56 [0.51,0.60]*** | 0.95 [0.82,1.10] | 0.76 [0.68,0.84]*** | 0.85 [0.73,1.00]* | 0.55 [0.49,0.62]*** |
| Marital Status |  |  |  |  |  |
| Married | [Ref] | [Ref] | [Ref] | [Ref] | [Ref] |
| Widowed | 1.05 [1.00,1.10] | 1.05 [0.92,1.21] | 1.14 [1.04,1.26]** | 1.51 [1.29,1.76]*** | 1.00 [0.92,1.08] |
| Divorced | 1.10 [1.05,1.15]*** | 1.22 [1.10,1.34]*** | 1.29 [1.21,1.38]*** | 1.65 [1.47,1.84]*** | 1.10 [1.04,1.17]** |
| Separated | 1.20 [1.08,1.33]** | 1.33 [1.09,1.63]** | 1.30 [1.07,1.57]** | 1.55 [1.19,2.02]** | 1.03 [0.90,1.19] |
| Never Married | 1.14 [1.07,1.21]*** | 0.98 [0.87,1.10] | 1.56 [1.42,1.70]*** | 1.71 [1.51,1.94]*** | 0.96 [0.88,1.04] |
| Living with Partner | 1.16 [1.06,1.26]** | 1.39 [1.20,1.60]*** | 1.34 [1.19,1.52]*** | 1.43 [1.21,1.70]*** | 1.12 [1.01,1.23]* |
| Poverty Category |  |  |  |  |  |
| Negative (<100%) | 3.06 [2.89,3.25]*** | 2.98 [2.57,3.46]*** | 1.11 [1.00,1.23] | 1.32 [1.14,1.53]*** | 2.59 [2.35,2.84]*** |
| Near Poor (100-125%) | 2.86 [2.65,3.07]*** | 3.75 [3.16,4.46]*** | 1.00 [0.86,1.17] | 1.23 [1.00,1.51] | 3.21 [2.90,3.55]*** |
| Low Income (125-200%) | 2.35 [2.21,2.49]*** | 3.19 [2.76,3.68]*** | 1.13 [1.03,1.24]* | 1.34 [1.16,1.54]*** | 2.73 [2.52,2.97]*** |
| Middle Income (200-400%) | 1.72 [1.63,1.81]*** | 2.34 [2.08,2.64]*** | 1.10 [1.02,1.19]* | 1.16 [1.03,1.30]* | 2.14 [1.99,2.30]*** |
| High Income (400%+) | [Ref] | [Ref] | [Ref] | [Ref] | [Ref] |
| Have Health Insurance | 1.27 [1.16,1.39]*** | 0.32 [0.29,0.35]*** | 0.29 [0.27,0.32]*** | 0.20 [0.18,0.22]*** | 0.52 [0.49,0.55]*** |
| Region |  |  |  |  |  |
| Northeast | [Ref] | [Ref] | [Ref] | [Ref] | [Ref] |
| Midwest | 0.96 [0.90,1.03] | 0.32 [0.29,0.35]*** | 1.16 [1.05,1.27]** | 1.39 [1.17,1.64]*** | 0.97 [0.88,1.06] |
| South | 1.08 [1.01,1.14]* | 0.32 [0.29,0.35]*** | 1.00 [0.91,1.09] | 1.44 [1.24,1.68]*** | 1.09 [1.00,1.19]* |
| West | 0.99 [0.93,1.05] | 0.32 [0.29,0.35]*** | 1.39 [1.27,1.53]*** | 1.37 [1.16,1.62]*** | 0.97 [0.89,1.06] |
| 2013 NCHS Urban-Rural Classification |  |  |  |  |  |
| Large Central Metro | [Ref] | [Ref] | [Ref] | [Ref] | [Ref] |
| Large Fringe Metro | 0.92 [0.87,0.96]** | 1.07 [0.95,1.19] | 1.12 [1.03,1.21]** | 0.89 [0.78,1.01] | 0.99 [0.93,1.07] |
| Medium and Small Metro | 0.98 [0.93,1.03] | 1.05 [0.94,1.17] | 1.00 [0.92,1.08] | 0.93 [0.81,1.06] | 0.95 [0.88,1.02] |
| Nonmetropolitan | 1.03 [0.98,1.10] | 0.99 [0.88,1.12] | 1.01 [0.91,1.12] | 0.76 [0.65,0.90]** | 0.94 [0.86,1.03] |
| Survey Year |  |  |  |  |  |
| 2019 | [Ref] | [Ref] | [Ref] | [Ref] | [Ref] |
| 2020 | 0.96 [0.91,1.02] | 0.87 [0.77,0.99]* | 1.19 [1.08,1.31]** | 0.96 [0.83,1.11] | 0.91 [0.84,0.98]* |
| 2021 | 0.95 [0.91,1.00] | 0.86 [0.77,0.96]** | 1.33 [1.22,1.44]*** | 0.97 [0.86,1.10] | 0.87 [0.82,0.92]*** |
| 2022 | 0.99 [0.94,1.03] | 0.74 [0.66,0.83]*** | 1.12 [1.03,1.22]** | 1.00 [0.88,1.10] | 0.86 [0.80,0.91]*** |
| 2023 | 1.02 [0.97,1.06] | 0.81 [0.73,0.90]*** | 1.04 [0.95,1.13] | 1.08 [0.96,1.22] | 0.81 [0.76,0.87]*** |

Source: Authors’ analysis of data from the National Health Interview Survey, 2019-2023

Notes: Adjusted Modified Poisson Regression Models. Decimals are rounded to the nearest hundredths place. All results are survey weighted.

† Reference Category: somewhat worried or not at all worried.

*p<0.05, **p<0.01, ***p<0.001

Appendix Table A2. Association between Fair or Poor Health and Health Care Access Problems and the Sociodemographic Characteristics of the Older Adults (50-64 Years)

| Outcome | Fair/Poor Health | Delayed medical care due to cost | No doctor visit within past year | No usual place to go for care | Very worried about paying medical bills if sick or in accident† |
| --- | --- | --- | --- | --- | --- |
|  | Prevalence Rate [CI] | Prevalence Rate [CI] | Prevalence Rate [CI] | Prevalence Rate [CI] | Prevalence Rate [CI] |
| Sociodemographic Characteristics |  |  |  |  |  |
| Female | 0.98 [0.93,1.04] | 1.36 [1.25,1.48]*** | 0.65 [0.61,0.70]*** | 0.55 [0.50,0.60]*** | 1.22 [1.15,1.29]*** |
| Race |  |  |  |  |  |
| White Non Hispanic | [Ref] | [Ref] | [Ref] | [Ref] | [Ref] |
| Black Non Hispanic | 1.06 [0.98,1.14] | 0.79 [0.69,0.90]*** | 0.64 [0.56,0.73]*** | 0.59 [0.49,0.71]*** | 1.09 [1.00,1.19] |
| Hispanic | 1.15 [1.08,1.22] | 0.85 [0.72,1.01] | 0.93 [0.83,1.04] | 0.90 [0.75,1.09] | 1.30 [1.17,1.44]*** |
| Asian Non Hispanic | 0.98 [0.87,1.10] | 0.51 [0.35,0.73]*** | 1.17 [0.98,1.39] | 0.98 [0.75,1.30] | 0.89 [0.76,1.05] |
| Other/Multi-race Non Hispanic | 1.22 [1.08,1.39]* | 0.92 [0.67,1.27] | 0.92 [0.74,1.15] | 0.94 [0.71,1.25] | 1.05 [0.86,1.30] |
| Education |  |  |  |  |  |
| Less than High School | 1.31 [1.21,1.42]*** | 1.12 [0.97,1.30] | 0.88 [0.78,1.00]* | 1.06 [0.90,1.25] | 1.00 [0.92,1.09] |
| High School | [Ref] | [Ref] | [Ref] | [Ref] | [Ref] |
| Some College | 0.90 [0.84,0.96]** | 1.13 [1.02,1.26]* | 0.91 [0.84,1.00]* | 0.94 [0.83,1.07] | 0.95 [0.89,1.02] |
| Bachelors | 0.58 [0.52,0.64]*** | 0.94 [0.81,1.09] | 0.96 [0.86,1.06] | 0.94 [0.81,1.09] | 0.74 [0.67,0.81]*** |
| Advanced | 0.53 [0.46,0.62]*** | 0.94 [0.78,1.13] | 0.77 [0.68,0.88]*** | 0.89 [0.74,1.08] | 0.53 [0.46,0.60]*** |
| Marital Status |  |  |  |  |  |
| Married | [Ref] | [Ref] | [Ref] | [Ref] | [Ref] |
| Widowed | 1.20 [1.08,1.31]*** | 1.09 [0.91,1.31] | 1.11 [0.96,1.30] | 1.59 [1.27,1.99]*** | 1.00 [0.89,1.12] |
| Divorced | 1.12 [1.05,1.19]** | 1.16 [1.04,1.30]* | 1.21 [1.11,1.31]*** | 1.54 [1.35,1.76]*** | 1.11 [1.03,1.19]** |
| Separated | 1.26 [1.11,1.42]*** | 1.30 [1.05,1.61]* | 1.24 [1.00,1.55] | 1.42 [1.08,1.88]* | 1.09 [0.94,1.28] |
| Never Married | 1.14 [1.05,1.23]** | 0.91 [0.80,1.04] | 1.41 [1.28,1.56]*** | 1.53 [1.32,1.76]*** | 0.95 [0.87,1.04] |
| Living with Partner | 1.19 [1.07,1.33]** | 1.34 [1.15,1.56]*** | 1.32 [1.16,1.51]*** | 1.35 [1.11,1.63]** | 1.13 [1.02,1.25]* |
| Poverty Category |  |  |  |  |  |
| Negative (<100%) | 4.23 [3.86,4.63]*** | 2.83 [2.39,3.35]*** | 1.00 [0.88,1.13] | 1.25 [1.03,1.50]* | 2.25 [2.01,2.52]*** |
| Near Poor (100-125%) | 4.27 [3.86,4.63]*** | 3.45 [2.86,4.17]*** | 0.99 [0.82,1.19] | 1.08 [0.84,1.40] | 2.92 [2.59,3.30]*** |
| Low Income (125-200%) | 3.30 [3.02,3.63]*** | 3.01 [2.55,3.56]*** | 1.13 [1.01,1.26]* | 1.36 [1.14,1.62]** | 2.56 [2.32,2.82]*** |
| Middle Income (200-400%) | 2.15 [1.97,2.34]*** | 2.38 [2.07,2.72]*** | 1.14 [1.05,1.25]** | 1.20 [1.05,1.37]** | 2.21 [2.03,2.40]*** |
| High Income (400%+) | [Ref] | [Ref] | [Ref] | [Ref] | [Ref] |
| Have Health Insurance | 1.40 [1.30,1.55]*** | 0.31 [0.28,0.35]*** | 0.29 [0.27,0.31]*** | 0.20 [0.18,0.22]*** | 0.51 [0.47,0.54]*** |
| Region |  |  |  |  |  |
| Northeast | [Ref] | [Ref] | [Ref] | [Ref] | [Ref] |
| Midwest | 0.97 [0.89,1.07] | 1.14 [0.97,1.34] | 1.11 [1.00,1.24] | 1.43 [1.18,1.75]*** | 1.01 [0.91,1.12] |
| South | 1.11 [1.03,1.20]** | 1.30 [1.11,1.52]** | 0.96 [0.87,1.07] | 1.42 [1.19,1.70]*** | 1.11 [1.00,1.22]* |
| West | 1.02 [0.93,1.11] | 1.17 [0.98,1.39] | 1.29 [1.16,1.43]*** | 1.29 [1.06,1.57]* | 0.98 [0.88,1.09] |
| 2013 NCHS Urban-Rural Classification |  |  |  |  |  |
| Large Central Metro | [Ref] | [Ref] | [Ref] | [Ref] | [Ref] |
| Large Fringe Metro | 0.94 [0.87,1.02] | 1.09 [0.96,1.25] | 1.11 [1.01,1.22]* | 0.90 [0.78,1.04] | 1.02 [0.93,1.11] |
| Medium and Small Metro | 0.97 [0.91,1.04] | 1.04 [0.92,1.18] | 0.99 [0.90,1.09] | 0.90 [0.78,1.05] | 0.97 [0.90,1.05] |
| Nonmetropolitan | 1.03 [0.95,1.12] | 0.99 [0.86,1.14] | 0.99 [0.88,1.11] | 0.75 [0.61,0.91]** | 0.95 [0.85,1.05] |
| Survey Year |  |  |  |  |  |
| 2019 | [Ref] | [Ref] | [Ref] | [Ref] | [Ref] |
| 2020 | 0.96 [0.88,1.05] | 0.89 [0.78,1.03] | 1.18 [1.05,1.32]** | 0.98 [0.82,1.16] | 0.92 [0.84,1.01] |
| 2021 | 0.97 [0.90,1.04] | 0.89 [0.79,1.01] | 1.29 [1.17,1.42]*** | 0.98 [0.85,1.13] | 0.89 [0.82,0.95]** |
| 2022 | 1.02 [0.95,1.10] | 0.73 [0.64,0.83]*** | 1.12 [1.02,1.23]* | 1.02 [0.88,1.19] | 0.88 [0.82,0.95]** |
| 2023 | 1.06 [0.98,1.14] | 0.84 [0.74,0.95]** | 1.05 [0.95,1.16] | 1.11 [0.97,1.27] | 0.83 [0.77,0.90]*** |

Source: Authors’ analysis of data from the National Health Interview Survey, 2019-2023

Notes: Adjusted Modified Poisson Regression Models. Decimals are rounded to the nearest hundredths place. All results are survey weighted.

† Reference Category: somewhat worried or not at all worried.

*p<0.05, **p<0.01, ***p<0.001

Appendix Table A3. Association between Fair or Poor Health and Health Care Access Problems and the Sociodemographic Characteristics of the Older Adults (65 – 85+ Years)

| Outcome | Fair/Poor Health | Delayed medical care due to cost | No doctor visit within past year | No usual place to go for care | Very worried about paying medical bills if sick or in accident† |
| --- | --- | --- | --- | --- | --- |
|  | Prevalence Rate [CI] | Prevalence Rate [CI] | Prevalence Rate [CI] | Prevalence Rate [CI] | Prevalence Rate [CI] |
| Sociodemographic Characteristics |  |  |  |  |  |
| Female | 0.85 [0.82,0.89]*** | 1.04 [0.90,1.21] | 0.81 [0.73,0.91]*** | 0.66 [0.57,0.77]*** | 1.11 [1.02,1.21]* |
| Race |  |  |  |  |  |
| White Non Hispanic | [Ref] | [Ref] | [Ref] | [Ref] | [Ref] |
| Black Non Hispanic | 1.21 [1.13,1.30]*** | 1.08 [0.84,1.39] | 0.72 [0.58,0.89]** | 0.69 [0.52,0.91]** | 1.54 [1.35,1.76]*** |
| Hispanic | 1.25 [1.15,1.37]*** | 1.29 [0.89,1.85] | 0.89 [0.72,1.10] | 0.65 [0.45,0.94]* | 1.79 [1.54,2.07]*** |
| Asian Non Hispanic | 1.04 [0.90,1.20] | 0.80 [0.47,1.35] | 1.12 [0.85,1.47] | 0.92 [0.54,1.58] | 1.26 [0.98,1.62] |
| Other/Multi-race Non Hispanic | 1.16 [1.00,1.36] | 1.40 [0.93,2.12] | 1.04 [0.74,1.48] | 1.71 [1.08,2.70]* | 1.13 [0.82,1.54] |
| Education |  |  |  |  |  |
| Less than High School | 1.28 [1.20,1.36]*** | 1.07 [0.83,1.37] | 1.04 [0.87,1.25] | 1.19 [0.90,1.57] | 1.23 [1.08,1.39]** |
| High School | [Ref] | [Ref] | [Ref] | [Ref] | [Ref] |
| Some College | 0.87 [0.82,0.91]*** | 0.91 [0.76,1.09] | 0.87 [0.76,0.99]* | 0.96 [0.80,1.16] | 0.88 [0.79,0.98]* |
| Bachelors | 0.62 [0.57,0.67]*** | 1.01 [0.80,1.28] | 0.93 [0.80,1.09] | 1.07 [0.87,1.32] | 0.87 [0.75,1.00] |
| Advanced | 0.56 [0.51,0.61]*** | 0.97 [0.72,1.31] | 0.72 [0.60,0.87]** | 0.78 [0.60,1.00] | 0.64 [0.53,0.78]*** |
| Marital Status |  |  |  |  |  |
| Married | [Ref] | [Ref] | [Ref] | 1.00 [Ref] | 1.00 [Ref] |
| Widowed | 1.04 [0.98,1.10] | 1.08 [0.87,1.34] | 1.17 [1.01,1.36]* | 1.44 [1.18,1.76]*** | 0.98 [0.87,1.11] |
| Divorced | 1.07 [1.00,1.13]* | 1.42 [1.17,1.72]** | 1.53 [1.33,1.76]*** | 1.90 [1.58,2.27]*** | 1.07 [0.95,1.20] |
| Separated | 1.01 [0.85,1.20] | 1.58 [0.97,2.58] | 1.59 [1.05,2.40]* | 2.22 [1.15,4.29]* | 0.90 [0.65,1.24] |
| Never Married | 1.10 [1.00,1.20] | 1.34 [1.02,1.78]* | 2.17 [1.82,2.57]*** | 2.47 [1.96,3.12]*** | 1.01 [0.86,1.18] |
| Living with Partner | 1.07 [0.94,1.21] | 1.62 [1.15,2.28]** | 1.33 [0.97,1.81] | 1.77 [1.23,2.55]** | 1.01 [0.79,1.29] |
| Poverty Category |  |  |  |  |  |
| Negative (<100%) | 2.12 [1.96,2.30]*** | 3.71 [2.75,5.01]*** | 1.51 [1.25,1.83]*** | 1.62 [1.26,2.09]*** | 3.73 [3.18,4.39]*** |
| Near Poor (100-125%) | 1.90 [1.73,2.09]*** | 4.89 [3.45,6.91] *** | 1.05 [0.82,1.34] | 1.61 [1.16,2.24]** | 4.18 [3.51,4.99]*** |
| Low Income (125-200%) | 1.70 [1.58,1.84]*** | 3.97 [3.00,5.26]*** | 1.15 [0.96,1.37] | 1.30 [1.03,1.65]* | 3.38 [2.91,3.92]*** |
| Middle Income (200-400%) | 1.36 [1.27,1.45]*** | 2.38 [1.86,3.04]*** | 1.02 [0.90,1.17] | 1.08 [0.87,1.33] | 2.14 [1.86,2.46]*** |
| High Income (400%+) | [Ref] | [Ref] | [Ref] | [Ref] | [Ref] |
| Have Health Insurance | 1.02 [0.84,1.24] | 0.30 [0.22,0.41]*** | 0.28 [0.22,0.37]*** | 0.20 [0.15,0.27]*** | 0.46 [0.39,0.56] |
| Region |  |  |  |  |  |
| Northeast | [Ref] | [Ref] | [Ref] | [Ref] | [Ref] |
| Midwest | 0.95 [0.88,1.03] | 0.94 [0.74,1.20] | 1.32 [1.12,1.56]** | 1.27 [0.96,1.67] | 0.88 [0.77,1.01] |
| South | 1.05 [0.98,1.12] | 1.05 [0.85,1.29] | 1.10 [0.94,1.30] | 1.51 [1.18,1.93]** | 1.06 [0.94,1.20] |
| West | 0.96 [0.89,1.03] | 1.10 [0.86,1.40] | 1.73 [1.46,2.05]*** | 1.56 [1.19,2.03]** | 0.97 [0.85,1.11] |
| 2013 NCHS Urban-Rural Classification |  |  |  |  |  |
| Large Central Metro | [Ref] | [Ref] | [Ref] | [Ref] | [Ref] |
| Large Fringe Metro | 0.90 [0.85,0.96]** | 0.99 [0.80,1.23] | 1.14 [0.98,1.32] | 0.86 [0.69,1.07] | 0.97 [0.86,1.10] |
| Medium and Small Metro | 0.98 [0.93,1.04] | 1.09 [0.89,1.34] | 1.04 [0.91,1.19] | 1.01 [0.81,1.25] | 0.91 [0.81,1.02] |
| Nonmetropolitan | 1.04 [0.97,1.12] | 0.99 [0.78,1.24] | 1.08 [0.89,1.30] | 0.80 [0.62,1.03] | 0.91 [0.80,1.04] |
| Survey Year |  |  |  |  |  |
| 2019 | [Ref] | [Ref] | [Ref] | [Ref] | [Ref] |
| 2020 | 0.96 [0.89,1.03] | 0.81 [0.64,1.03] | 1.23 [1.02,1.47]* | 0.93 [0.72,1.20] | 0.87 [0.76,1.00]* |
| 2021 | 0.94 [0.88,1.00] | 0.75 [0.60,0.93]* | 1.45 [1.25,1.68]*** | 0.94 [0.77,1.16] | 0.84 [0.74,0.95]** |
| 2022 | 0.96 [0.91,1.03] | 0.77 [0.62,0.97]* | 1.13 [0.96,1.32] | 0.93 [0.75,1.16] | 0.80 [0.71,0.90]*** |
| 2023 | 1.00 [0.94,1.05] | 0.73 [0.59,0.90]** | 1.02 [0.87,1.19] | 1.03 [0.83,1.27] | 0.79 [0.70,0.89]*** |

Source: Authors’ analysis of data from the National Health Interview Survey, 2019-2023

Notes: Adjusted Modified Poisson Regression Models. Decimals are rounded to the nearest hundredths place. All results are survey weighted.

† Reference Category: somewhat worried or not at all worried.

*p<0.05, **p<0.01, ***p<0.001
