# Supplementary material for: The need to protect older immigrants’ health in a changing policy landscape
Source: Health Aff Sch. 2026 May 23;4(6):qxag127. doi: 10.1093/haschl/qxag127 (PMC13268766; doi:10.1093/haschl/qxag127)
Supplement: qxag127_Supplementary_Data [file qxag127_supplementary_data.zip › coi_disclosure_Mervin Dino.docx]

| ICMJE DISCLOSURE FORM | |
| --- | --- |
| **Date:** | 5/5/2026 |
| **Your Name:** | Mervin Dino |
| **Manuscript Title:** | The Need to Protect Older Immigrants' Health in a Changing Policy Landscape |
| **Manuscript Number (if known):** | ID HASCHOLAR-D-25-00552 |
| In the interest of transparency, we ask you to disclose all relationships/activities/interests listed below that are related to the content of your manuscript. “Related” means any relation with for-profit or not-for-profit third parties whose interests may be affected by the content of the manuscript. Disclosure represents a commitment to transparency and does not necessarily indicate a bias. If you are in doubt about whether to list a relationship/activity/interest, it is preferable that you do so.  The author’s relationships/activities/interests should be defined broadly. For example, if your manuscript pertains to the epidemiology of hypertension, you should declare all relationships with manufacturers of antihypertensive medication, even if that medication is not mentioned in the manuscript.  In item #1 below, report all support for the work reported in this manuscript without time limit. For all other items, the time frame for disclosure is the past 36 months. | |

|  | | | **Name all entities with whom you have this relationship or indicate none (add rows as needed)** | **Specifications/Comments (e.g., if payments were made to you or to your institution)** |
| --- | --- | --- | --- | --- |
| **Time frame: Since the initial planning of the work** | | | | |
| **1** | All support for the present manuscript (e.g., funding, provision of study materials, medical writing, article processing charges, etc.)  **No time limit for this item.** | | \|  \| **None** \| \| --- \| --- \|  \|  \|  \| \| --- \| --- \| \|  \|  \| \|  \| Click the tab key to add additional rows. \| | |
| **Time frame: past 36 months** | | | | |
| **2** | | Grants or contracts from any entity (if not indicated in item #1 above). | \|  \| **None** \| \| --- \| --- \|  \| U.S. Centers for Disease Control and Prevention \| Through my employment at the City of Chicago (Chicago Department of Public Health), I serve as the Principal Investigator/Project Director for the CDC Health Disparities Grant (OT21-2103: National Initiative to Address COVID-19 Health Disparities Among Populations at High-Risk and Underserved, Including Racial and Ethnic Minority Populations and Rural Communities). Funding is provided to the Chicago Department of Public Health. \| \| --- \| --- \| \| U.S. Department of Housing and Urban Development \| Through my employment at the City of Chicago (Chicago Department of Public Health), I support grant activities under the HUD Lead Hazard Reduction and HUD Healthy Homes Production Grant Programs, with funding provided to the Chicago Department of Public Health. \| \|  \|  \| \| Blue Cross Blue Shield of Illinois \| Through my employment at the City of Chicago (Chicago Department of Public Health), I support grant activities under a philanthropy grant from Blue Cross Blue Shield of Illinois provided to the Chicago Department of Public Health. \| | |
| **3** | | Royalties or licenses | \|  \| **None** \| \| --- \| --- \|  \|  \|  \| \| --- \| --- \| \|  \|  \| \|  \|  \| | |
| **4** | | Consulting fees | \|  \| **None** \| \| --- \| --- \|  \|  \|  \| \| --- \| --- \| \|  \|  \| \|  \|  \| \|  \|  \| | |
| **5** | | Payment or honoraria for lectures, presentations, speakers bureaus, manuscript writing or educational events | \|  \| **None** \| \| --- \| --- \|  \|  \|  \| \| --- \| --- \| \|  \|  \| \|  \|  \| | |
| **6** | | Payment for expert testimony | \|  \| **None** \| \| --- \| --- \|  \|  \|  \| \| --- \| --- \| \|  \|  \| \|  \|  \| | |
| **7** | | Support for attending meetings and/or travel | \|  \| **None** \| \| --- \| --- \|  \| de Beaumont Foundation \| I disclose receiving a third-party sponsorship for travel expenses for the 40 Under 40 in Public Health Leadership Summit from the de Beaumont Foundation. \| \| --- \| --- \| \|  \|  \| \|  \|  \| | |
| **8** | | Patents planned, issued or pending | \|  \| **None** \| \| --- \| --- \|  \|  \|  \| \| --- \| --- \| \|  \|  \| \|  \|  \| | |
| **9** | | Participation on a Data Safety Monitoring Board or Advisory Board | \|  \| **None** \| \| --- \| --- \|  \|  \|  \| \| --- \| --- \| \|  \|  \| \|  \|  \| | |
| **10** | | Leadership or fiduciary role in other board, society, committee or advocacy group, paid or unpaid | \|  \| **None** \| \| --- \| --- \|  \|  \|  \| \| --- \| --- \| \|  \|  \| \|  \|  \| | |
| **11** | | Stock or stock options | \|  \| **None** \| \| --- \| --- \|  \|  \|  \| \| --- \| --- \| \|  \|  \| \|  \|  \| | |
| **12** | | Receipt of equipment, materials, drugs, medical writing, gifts or other services | \|  \| **None** \| \| --- \| --- \|  \|  \|  \| \| --- \| --- \| \|  \|  \| \|  \|  \| | |
| **13** | | Other financial or non-financial interests | \|  \| **None** \| \| --- \| --- \|  \| Chicago Department of Public Health \| I am an employee of the City of Chicago (Chicago Department of Public Health), where I serve as Assistant Commissioner for Health Protection. My contributions to the manuscript are through the Johns Hopkins Bloomberg School of Public Health and do not reflect the views of the City of Chicago. \| \| --- \| --- \| \|  \|  \| \|  \|  \| | |
|  | |  |  | |
| **Please place an “X” next to the following statement to indicate your agreement:** | | | | |
|  | | I certify that I have answered every question and have not altered the wording of any of the questions on this form. | | |
